# The effect of topical and intraurethral alprostadil on erectile function: A systematic review and meta‐analysis

**DOI:** 10.1111/andr.70025

**Published:** 2025-03-19

**Authors:** Iason Papadopoulos, Maksim Tishukov, Ioannis Sokolakis, Ioannis Katafigiotis, Ioannis Leotsakos, Markos Karavitakis, Julian Marcon, Christian G Stief, Nikolaos Pyrgidis

**Affiliations:** ^1^ Department of Urology University Hospital LMU Munich Munich Germany; ^2^ Institute for Medical Information Processing Biometry and Epidemiology (IBE) Faculty of Medicine LMU Munich Germany; ^3^ Pettenkofer School of Public Health Munich Germany; ^4^ School of Medicine Faculty of Health Sciences Aristotle University of Thessaloniki Thessaloniki Greece; ^5^ Second Department of Urology Medical School Aristotle University of Thessaloniki Thessaloniki Greece; ^6^ Department of Laparoscopy and Endourology Central Urology Lefkos Stavros the Athens Clinic Athens Greece

**Keywords:** alprostadil, erectile dysfunction, intraurethral, safety, topical, treatment

## Abstract

**Background:**

Erectile dysfunction (ED) affects up to 50% of men over 40. While phosphodiesterase‐5 inhibitors (PDE5i) are the first line of medical treatment, they are not always effective. Alprostadil, available in injectable, topical, and intraurethral forms, offers an alternative treatment modality.

**Objectives:**

This systematic review and meta‐analysis aim to evaluate the efficacy and safety of topical and intraurethral forms of alprostadil.

**Materials and methods:**

The objectives and methods of this systematic review and meta‐analysis were predefined in a protocol registered on PROSPERO (CRD42021260894). We systematically searched PubMed, EMBASE, and the Cochrane Library up to April 2024. Using a random‐effects model, we compared the efficacy of topical and intraurethral forms of alprostadil against placebo. Additionally, we performed a qualitative assessment of the studies using RoB‐2 and ROBINS‐I tools.

**Results:**

The analysis included 11 randomized controlled trials and 4 non‐randomized studies, encompassing 5869 patients with a mean age of 60 ± 9.4 years. The meta‐analysis showed a statistically significant improvement in the IIEF score by 4.7 points (95% CI: 2.4–7.1, *I^2^* = 97%) in patients using the topical form of alprostadil compared to placebo. There was also a statistically significant improvement of ED in patients using intraurethral alprostadil compared to placebo, with a pooled odds ratio of 0.08 (95% CI: 0.04–0.16, *I* ^2^ = 54%). The most common adverse events reported were penile pain and erythema.

**Discussion:**

These results are limited by the variability in study designs and the relatively small number of included studies in the meta‐analysis. Moreover, the low methodological quality of the included studies further limits the strength of the conclusions.

**Conclusions:**

Topical and intraurethral alprostadil significantly improve ED symptoms compared to placebo and are generally safe, with no serious adverse events. Further trials are necessary to confirm and expand on these findings.

## 1 INTRODUCTION

Erectile dysfunction (ED) is characterized by the persistent inability to achieve and maintain an erection sufficient for satisfactory sexual performance.^1^ This condition not only impairs the quality of life of the affected individuals but also impacts their partners.^2^ Available evidence suggest that it negatively affects up to 50% of male adults over the age of 40 to some degree.^3^, ^4^ Treatment options depend on the cause of ED and range from lifestyle changes to oral pharmacotherapy and surgical interventions.^5^ Of them, phosphodiesterase‐5 inhibitors (PDE5i) are considered the most favored treatment.^6^ Nevertheless, a significant number of patients experience dissatisfaction, demonstrate low adherence rates, or discontinue first‐line ED treatments due to inadequate efficacy, inconvenient administration, adverse events (AEs), or contraindications.^7^, ^8^

Alprostadil, a synthetic formulation of prostaglandin E1, represents an alternative treatment modality that has shown efficacy in patients who do not respond to PDE5i pharmacotherapy.^9^ It is available in intracavernosal injections, topical applications and intraurethral suppositories.^10^ While intracavernosal injections are highly effective, they lead to relatively high dropout rates of up to 40%–50% due to their unconventional way of administration.^11^, ^12^ Conversely, the less invasive topical and intraurethral routes are preferred by some patients due to their simplicity and enhanced safety profile compared to other available ED treatments.^10^, ^13^

Despite the widespread use of alprostadil in clinical practice, there is a lack of comprehensive, cumulative evidence assessing its effectiveness and safety. To address this gap, we conducted, the first to our knowledge, systematic review and meta‐analysis exploring the efficacy and safety of intraurethral and topical alprostadil compared to placebo or other treatment modalities across various subgroups of patients with ED.

## 2 MATERIALS AND METHODS

### 2.1 Data Sources and Searches

We established predefined objectives and methods in a protocol registered at PROSPERO (CRD42021260894) and adhered to the guidelines in the Cochrane Handbook for Systematic Reviews of Interventions and the PRISMA statement.^14^, ^15^ Our literature search covered PubMed, EMBASE, and the Cochrane Library databases from inception until April 2024. Additionally, we manually searched significant sources of grey literature, including clinical trial registries and conference abstracts published in major urology and sexual medicine journals. We also performed forward and backward citation searches by examining the reference lists of all included studies. The detailed search syntax and search strings are available in Supporting Information 1.

### 2.2 Selection Criteria

We included randomized controlled trials (RCTs) and prospective interventional studies (non‐RCTs) that assessed the effect of topical or intraurethral alprostadil versus placebo or other treatment modalities in male adults with ED of vasculogenic, post‐surgical or mixed aetiology. Only comparative human studies published in any language that evaluated changes from baseline in self‐reported erectile function using validated questionnaires were considered. Excluded were studies comparing combination therapy versus placebo, studies without a comparator arm, and articles evaluating psychosocial and behavioral interventions for ED of mixed aetiology. We also excluded studies assessing the role of alprostadil in diagnosing ED using duplex Doppler ultrasound and studies focusing on patients with neurogenic ED or spinal cord injury‐associated ED. In cases of multiple records with potentially overlapping populations, only the most recent study was included.

### 2.3 Data Extraction and Risk of Bias/Quality Assessment

**FIGURE 1:**
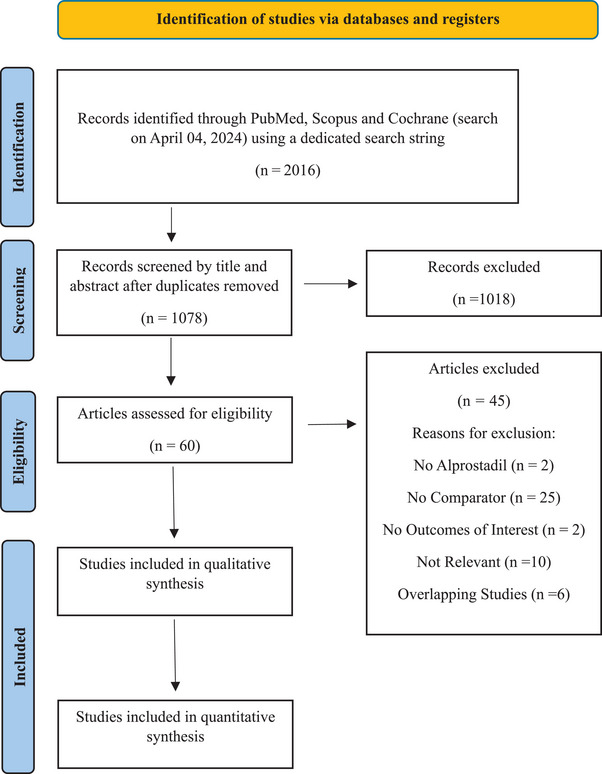
Preferred reporting items for systemic reviews and meta‐analyses (PRISMA) flow sheet for study selection.

Two independent authors (Iason Papadopoulos and Nikolaos Pyrgidis) screened the titles and abstracts of all retrieved records. Full texts of potentially eligible articles were evaluated based on the selection criteria. Data extraction was independently conducted by two authors (Iason Papadopoulos and Maksim Tishukov) using a predesigned Microsoft Excel spreadsheet. For each included study, we recorded data on study and participant characteristics, as well as efficacy and safety outcomes of topical or intraurethral alprostadil versus placebo or other treatment modalities. Disagreements between the authors were resolved by consensus. Figure 1 shows the flow chart of the Preferred Reporting Items for Systemic Reviews and Meta‐analyses (PRISMA) for the selection of the included studies.

For studies assessing erectile function at multiple time points, we extracted data from the baseline and the last evaluation only. In studies assessing different therapeutic doses, we used the mean data from all active doses. When the standard deviation for the mean erectile function change from baseline was not reported, it was calculated from the relevant standard error, confidence interval (CI), or *p*‐value.^16^ If insufficient data were available to calculate standard deviations, they were imputed from the correlation coefficient reported in other included trials. For AEs, we synthesized data on any AE reported in the included studies using an exploratory approach.

To evaluate the risk of bias within the studies, two authors (Iason Papadopoulos and Nikolaos Pyrgidis) independently used the RoB‐2 tool for RCTs^17^ and the Robins‐I tool for observational studies.^18^ Any disagreements were resolved once again by consensus between the authors.

### 2.4 Data Synthesis and Statistical Analysis

Given the limited number of relevant studies, we conducted an inverse variance random effects meta‐analysis, focussing on RCTs that compared topical or intraurethral treatments with placebo. We calculated weighted mean differences for changes in the mean International Index of Erectile Function (IIEF) and odds ratios for the persistence of symptoms post‐treatment with topical or intraurethral alprostadil compared to placebo, along with the corresponding 95% CIs. Separate analyses were performed for the mean IIEF change and the number of patients with persistent ED symptoms, on the basis of different active treatment modalities. Due to the limited number of included studies, we could not conduct subgroup or sensitivity analyses.

A meta‐analysis was conducted for an outcome when at least three studies used a similar treatment modality in both the active and control arms. Heterogeneity among the meta‐analytic effects was evaluated using the *I^2^* statistic, with its significance assessed via the *p*‐value of Cochran's *Q* test. Because of the small number of studies included in the meta‐analysis, we could not address potential publication bias. All statistical analyses were performed using R (version 4.4.1; R Core Team) with the “meta” package.

## 3 RESULTS

### 3.1 Study Results and Characteristics

Our systematic literature search initially identified 1078 unique studies. After screening titles and abstracts, 60 studies were deemed eligible for full‐text evaluation. Ultimately, 15 studies (11 RCTs and 4 non‐RCTs) with a total of 5869 participants were included in the qualitative synthesis.^19^, ^20^, ^21^, ^22^, ^23^, ^24^, ^25^, ^26^, ^27^, ^28^, ^29^, ^30^, ^31^, ^32^, ^33^ The mean patient age was 60 ± 9.4 years. The duration of treatment for participants ranged from 1 to 9 months. Thirteen studies focussed on patients with primarily organic ED,^19^, ^20^, ^21^, ^22^, ^23^, ^24^, ^26^, ^27^, ^28^, ^30^, ^31^, ^32^, ^33^ and two studies only considered patients with ED after radical prostatectomy.^25^, ^29^ The study selection process is illustrated in Supporting Information 2 and 3, and the characteristics of the included studies with AEs for all treatment modalities are documented in Tables 1 and 2.

**TABLE 1:** Characteristics of studies used in qualitative synthesis comparing topical alprostadil versus placebo or other treatment modalities.

Author, Year and Country	Type of Study	Active vs. Control	Assessment of Erectile Function	Number of Participants Active vs. Control	Mean Age ± SD	Number of Dropouts Active vs. Control	Duration of Treatment	Number of Adverse Events Active vs. Control	Type of Adverse Events
Abad 2021^19^ Spain	non‐RCT	Topical + PDE5I vs. Topical	IIEF‐5, SEP‐2, SEP‐3, GAQ‐1, GAQ‐2	98 vs. 72	59.2 ± 10	3 vs. 3	3 months	63 (64%) vs. 51 (71%)	Active: penile pain (17%) urethral discomfort (6%), headache (2%), dizziness (3%), back pain (1%), flushing (7%), priapism (0) Control: penile pain (24%), urethral discomfort (4%), dizziness (4%), priapism (0)
Buvat 2012^20^ Multinational	RCT	Topical vs. Placebo	IIEF‐EF, SEP‐2, SEP‐3, GAQ	NA	NA	NA	3 months	NA	NA
Goldstein 2001^21^ USA	RCT	Topical vs. Placebo	Erection scale (1‐5, but not EAS) and a protractor to measure the angle of erection.	31 vs. 29	NA	NA	2 office visits	15 (48%) vs. 9 (31%)	Minor to mild AEs reported in both groups: erythema, warmth, burning, stinging, coolness, heat, and tingling except for conjunctivitis (6%) and hypotension (3%), which were reported in the active group. No cases of priapism were reported in either group.
McVary 1999^22^ USA	non‐RCT	Topical vs. Placebo	O'Leary Brief Sexual Function Inventory, Erectile Response Scale (1–5)	36 vs. 12	57.4 ± 10.8	1 vs. 0	1 office visit	NA	Active: severe penile burning sensation (20%), penile pruritus (1 patient), Both active and control: warm sensation on application site (75–100%), light penile erythema (70–100%), priapism (0)
Padma‐Nathan 2003^23^ | Study 1 USA	RCT	Topical vs. Placebo	IIEF‐EF, SEP, PSAE, GAQ	121 vs. 40	56.5 ± 7.3	35 vs. 9	3‐4 office visits over several weeks	22 (18%) vs. 0	Active: urogenital pain (13%), hypotension (5%) Control: no AEs
Padma‐Nathan 2003^23^ | Study 2 USA	RCT	Topical vs. Placebo	IIEF‐EF, SEP, PSAE, GAQ	107 vs. 35	59.5 ± 8.5	0 vs. 0	3‐4 office visits over several weeks	15 (14%) vs. 0	Active: hypotension (6%), urogenital pain (6%), leg pain (1%), chest pain (1%), partner AEs (1%), priapism (0) Control: no AEs
Padma‐Nathan 2006^24^ USA	RCT	Topical vs. Placebo	IIEF‐EF, SEP‐2, SEP‐3, GAQ	1298 vs. 434	60.7 ± 9.8	60 vs. 23	4 months	757 (58%) vs. 54 (12%)	Active: penile burning (22%) genital pain (15%), penile erythema (9%), partner AEs (6%), headache (0.05%), priapism (0) Control: penile burning (0.5%) genital pain (15%), penile erythema (9%), partner AEs (6%), priapism (0)

Abbreviations: AEs: adverse events; EAS: erection assessment scale; GAQ: global assessment question; IIEF: international index of erectile function; IIEF‐EF: international index of erectile function—erectile function; NA: not available; PDE5i: phosphodiesterase 5 inhibitors; PSAE: patient self‐assessment of erection; RCT: randomized controlled trial; SD: standard deviation; SEP: sexual encounter profile.

**TABLE 2:** Characteristics of studies used in qualitative synthesis comparing intraurethral alprostadil versus placebo or other treatment modalities.

Author, Year and Country	Type of Study	Active vs. Control	Assessment of Erectile Function	Number of Participants Active vs Control	Mean Age ± SD	Number of Dropouts Active vs Control	Duration of Treatment	Number of Adverse Events Active vs Control	Type of Adverse Events
Belinski 2021^25^ Romania	non‐RCT	Intraurethral + PDE5i vs. PDE5i	IIEF‐5	55 vs. 56	66 ± 1.4	NA	6 Months	NA	NA
Shabsigh 2000^26^ USA	RCT—Crossover	Intracavernosal vs. Intraurethral	IIEF, Erection Scale (0‐4), Buckling Test	95 vs. 95	59.2 ± 10.6	27 vs. 27	1‐2 Months	48 (50%) vs. 26 (27%)	Active: application site reaction (2%) penile pain (34%), local bleeding (1%) prolonged erection (3%), priapism (0) Control: application site reaction (10%) penile pain (25%), local bleeding (3%), priapism (0)
Shokeir 1999^27^ Saudi Arabia	RCT	Intracavernosal vs. Intraurethral	EAS	30 vs. 30	55.5 ± 17.4	20 vs. 5	3 Months	14 (46%) vs. 5 (16%)	Active: urogenital pain (47%), priapism (0) Control: urogenital pain (7%), urethral bleeding (3%), dizziness (6%), priapism (0)
Coca 2020^28^ Romania	non‐RCT	Intraurethral vs. PDE5I	IIEF‐5, IELT, IIEF‐OS, GAQ	67 vs. 50	NA	NA	1 Month	NA	NA
McCullough 2010^29^ USA	Open‐label RCT	Intraurethral vs. PDE5I	IIEF‐EF, GAQ, SEP, EDITS, SPL	139 vs. 73	56.4 ± 6.3	42 vs. 14	9 Months	NA	Active: penile burning, penile discomfort, priapism (0) Control: headache and flushing (no numbers given), priapism (0)
Hellstrom 1996^30^ USA	RCT—Crossover	Intraurethral vs. Placebo	EAS, categorical and visual analogue scales, penile volume measurements.	68 vs. 68	58.6 ± 10.7	NA	2‐4 Weeks	14 (20.6%) vs. 0	Active: penile pain (up to 18%), dizziness (1%) sweating (1%), priapism (0) Control: no significant side effects reported, priapism (0)
Padma‐Nathan 1997^31^ USA	RCT	Intraurethral vs. Placebo	EAS	485 vs. 511	61.5 ± 8.3	24 vs. 11	3 Months	194 (40.00%) vs. 27 (5.28%)	Active: penile pain (33%), minor urethral trauma (5%), dizziness (2%), priapism (0) Control: penile pain (3%), minor urethral trauma (1%), urinary tract infections (1%), priapism (0)
Williams 1998^32^ Multinational	RCT	Intraurethral vs. Placebo	EAS	78 vs. 81	57.3 ± 8.6	11 vs. 8	3 Months	18 (23.08%) vs. 5 (6.17%)	Active: urethral pain/burning (6%), penile pain (5%), testicular pain (3%), minor urethral bleeding (1%), dizziness (3%), priapism (0) Control: penile pain (1%), minor urethral bleeding (1%), priapism (0)
Cai 2018^33^ Italy	RCT—Crossover	Intraurethral (NEW.AR) vs. Topical (ST.AR)	IIEF‐5, SEP‐2, SEP‐3, PSAE	71 vs. 71	59.7 ± 9	9 vs. 9	NA	64 vs. 60	Active: burning (45%), erythema (45%), priapism (0) Control: burning (35%), erythema (48%), priapism (0)

Abbreviations: EAS: erection assessment scale; EDITS: erectile dysfunction inventory of treatment satisfaction; GAQ: global assessment question; IELT: intravaginal ejaculation latency time; IIEF: International index of erectile function; IIEF‐OS: International index of erectile function—overall satisfaction; NA: not available; NEW.AR: new administration route; PDE5i: phosphodiesterase 5 inhibitors; PSAE: patient self‐assessment of erection; RCT: randomized controlled trial; SD: standard deviation; SEP: sexual encounter profile; SPL: stretched penile length; ST.AR: standard administration route.

The overall risk of bias was classified as “with some concerns” in five RCTs and “high” in four RCTs (Supporting Information 4). Among non‐RCTs, one was considered to have a moderate risk, while three exhibited a serious risk of bias (Supporting Information 5).^34^

### 3.2 Topical Alprostadil versus Placebo

**FIGURE 2:**
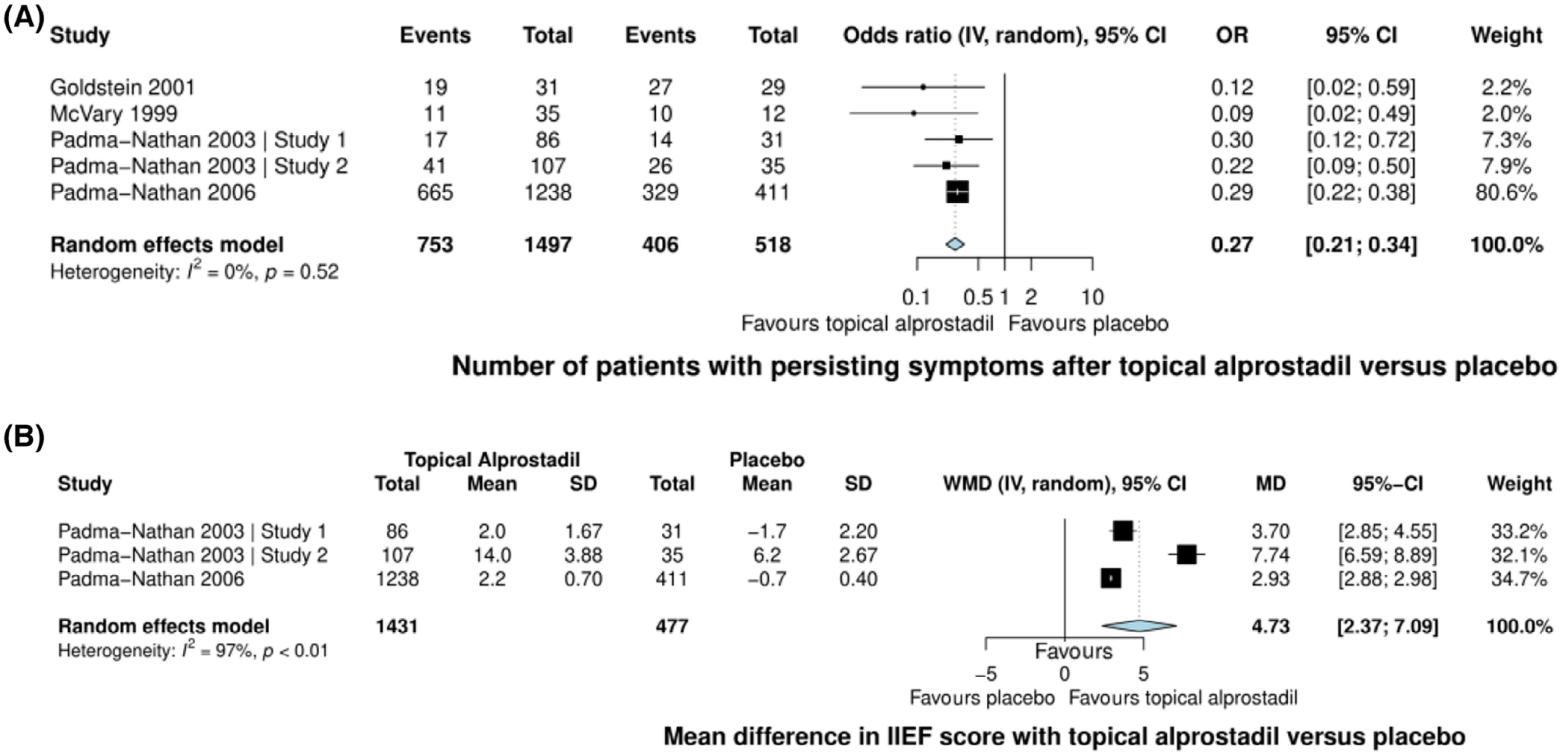
(A) Forest plot of the odds ratio in the number of patients with persisting erectile dysfunction symptoms after the use of topical alprostadil versus placebo. (B) Forest plot of the mean difference in IIEF score regarding topical alprostadil versus placebo. CI: confidence interval; IV: Inverse Variance; IIEF: International index of erectile function; MD: mean difference; OR: Odds Ratio; SD: standard deviation; WMD: weighted mean difference.

Six studies explored the efficacy of topical alprostadil versus placebo,^20^, ^21^, ^22^, ^23^, ^24^ with five ultimately included in the meta‐analysis,^21^, ^22^, ^23^, ^24^ encompassing a total of 2143 patients. Topical alprostadil significantly reduced the number of patients with persisting ED symptoms with an overall odds ratio of 0.27 (95% CI: 0.2–0.34, *I^2^* = 0%), compared to placebo (Figure 2A).

Additionally, in the meta‐analysis of three studies assessing IIEF scores before and after treatment,^23^, ^24^ topical alprostadil was significantly associated with an increase in the IIEF by 4.73 more points compared to placebo (95% CI: 2.37–7.09, *I^2^* = 97%, Figure 2B).

AEs were more frequent in the active treatment groups, with common effects such as penile burning (up to 22%), genital pain (up to 17%), and erythema (up to 9%). In contrast, the placebo groups reported significantly fewer AEs. For further details, refer to Table 1.

### 3.3 Topical Alprostadil versus Other Treatment Modalities

Abad et al. (2021) evaluated the efficacy of topical alprostadil alone compared to a combination of topical alprostadil and PDE5i in patients with ED who did not respond to PDE5i monotherapy. Their findings showed a significant improvement in erectile function after combination treatment without compromising safety. Specifically, patients receiving combination therapy reported an improvement in the mean IIEF‐5 score from 12.4 ± 3.4 to 17.1 ± 4.5 (*p* < 0.001). On the contrary, patients treated only with topical alprostadil did not show any significant changes, with the mean IIEF‐5 score slightly increasing from 12.2 ± 2.5 to 12.7 ± 3.1 (*p* = 0.15). The AEs reported in this study included systemic AEs such as headache, flushing, and dizziness, which can be attributed to the systemic action of PDE5i.^19^

Moreover, Cai et al. compared the topical versus intraurethral application of alprostadil. The results demonstrated that the intraurethral application significantly enhanced both drug efficacy and patient satisfaction. Specifically, intraurethral application led to significant improvement in the IIEF‐5 score and Sexual Encounter Profile (SEP) responses compared to topical application (IIEF‐5 improvement of 6.3 vs. 3.8 points; *p* < 0.001 and positive response to SEP, question 2: 27 vs. 10; *p* = 0.002, accordingly) The AEs reported were similar in form and frequency in both active and control groups with genital pain and erythema reported up to 35% and 48 accordingly.^33^

### 3.4 Intraurethral Alprostadil versus Placebo

**FIGURE 3:**
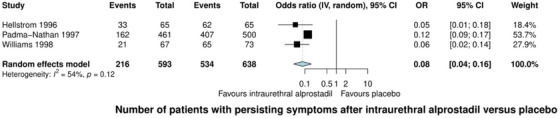
Forest plot of the odds ratio in the number of patients with persisting erectile dysfunction symptoms after the use of intraurethral alprostadil versus placebo. CI: confidence interval; IV: inverse variance; OR: odds ratio.

Three studies comparing intraurethral alprostadil to placebo were included in both qualitative and quantitative analyses.^30^, ^31^, ^32^ Intraurethral alprostadil significantly reduced ED symptoms compared to placebo, with a pooled odds ratio of 0.08 (95% CI: 0.04–0.16, *I^2^ *= 54%, Figure 3).^30^, ^31^, ^32^

The AEs in the active treatment group predominantly included penile pain (up to 33%), urethral burning (up to 6%), and minor urethral trauma (up to 5%). The placebo groups experienced fewer and less severe AEs, such as penile pain (up to 3%) and minor urethral trauma (1%). For more detailed summaries of AEs, we refer you to Table 2.

### 3.5 Intraurethral Alprostadil versus Other Treatment Modalities

Two studies compared intraurethral alprostadil to PDE5i. Coca et al. (2020) demonstrated improvements in both ED and premature ejaculation using intraurethral alprostadil compared to PDE5i monotherapy (mean IIEF improvement—intraurethral group: 6.84 ± 3.3, PDE5i monotherapy group: 8.3 ± 3.54, *p* = 0.5).^28^ McCullough et al. (2010), studying the recovery of erectile function after nerve‐sparing radical prostatectomy found no statistically significant differences in the IIEF scores between patients treated with intraurethral alprostadil and PDE5i.^29^

Belinski et al. (2021) evaluated the efficacy of combining intraurethral alprostadil with PDE5i compared to PDE5i monotherapy for penile rehabilitation following radical prostatectomy. Their study indicated that the combination therapy significantly enhances the likelihood of erectile function recovery to preoperative levels (Odds ratio: 3.6; 95% CI: 1.5–8.6).^25^

Finally, two further studies compared intracavernosal versus intraurethral alprostadil. Shokeir (1999) et al. found that 90% of patients receiving intracavernosal alprostadil achieved a good erectile response, defined as the ability to maintain an erection sufficient for vaginal penetration, compared to 60% in the intraurethral group (*p* < 0.05). After 3 months, intercourse was reported by 87% versus 53% (*p* < 0.05), though urogenital pain was higher in the intracavernosal group (47% vs. 7%, *p* < 0.05).^27^ Shabsigh et al. (2000) also validated the superior effectiveness of intracavernosal alprostadil. More specifically, their study demonstrated that 93% of patients achieved erections sufficient for intercourse with intracavernosal alprostadil versus 62% with intraurethral alprostadil (*p* < 0.0001). Patient and partner satisfaction were higher in the intracavernosal group, though penile pain was common in both treatments (34% vs. 25%).^26^

## 4 DISCUSSION

In this systematic review and meta‐analysis, we evaluated the efficacy as well as the safety of topical and intraurethral alprostadil for ED in comparison to placebo and other treatment modalities. Our findings indicate that both topical and intraurethral alprostadil significantly improve erectile function and reduce the incidence of persistent ED symptoms compared to placebo. Our systematic review has further revealed that the AEs associated with both treatment modalities are primarily localized to urogenital symptoms, such as penile burning and erythema. Additionally, urethral trauma, including bleeding and urinary tract infections, was only sporadically reported in patients receiving intraurethral alprostadil. Notably, in multiple studies, penile pain was the most reported symptom, affecting 7%–11% of patients using both topical and intraurethral alprostadil. Importantly, no cases of priapism were reported as an AE in any of the included studies using intraurethral or topical alprostadil. This finding is significant, as priapism is an important AE associated with intracavernosal alprostadil.

It should be highlighted that Williams et al. (1998) indicated that the number and the degree of AEs does not seem to correlate with the applied dosage of intraurethral alprostadil.^32^ Of note, topically applied alprostadil demonstrates a favourable safety profile compared to intraurethral alprostadil, with AEs occurring in about 3% of all cases.^35^ On the basis of previous notion, available evidence indicates that the intraurethral and topical forms of alprostadil have similar bioavailability. In particular, a study using gamma scintigraphy to track radiolabelled alprostadil found no significant differences in its absorption between intraurethral and topical application, although it did not assess the impact of the bioavailability on erectile function.^36^ Accordingly, the absence of systemic AEs after topical or intraurethral treatment may be attributed to the pharmacokinetic properties of alprostadil, which is rapidly metabolized, minimizing its systemic toxicity.^10^

Although PDE5i are typically the first‐line treatment modality for ED, up to 11%–40% of PDE5i users do not respond adequately.^37^ Additionally, systemic AEs, high therapy costs, and potential drug interactions render some patients unsuitable for this treatment modality.^38^ Topical and intraurethral alprostadil, although less effective than intracavernosal injections, have proven more effective than placebo in our meta‐analysis, providing a less invasive and potentially more attractive alternative.^10^, ^13^ The latter is supported by a study indicating that 53% of patients preferred topical alprostadil as a first‐line treatment for ED, compared to 42% who preferred oral pharmacotherapy.^39^ Interestingly, a meta‐analysis concluded that a combination therapy with PDE5i and alprostadil, regardless of the method of administration, provides a more effective therapeutic approach for ED recovery than when these treatments are used separately.^40^

While PDE5 inhibitors, such as sildenafil, rely on sexual desire to achieve an erection, alprostadil works through a different mechanism that does not require sexual stimulation.^41^ Alprostadil, a form of prostaglandin E1, uses cyclic adenosine monophosphate (cAMP) to directly relax smooth muscle and increase penile blood flow to the corpus cavernosum.^42^ This allows for an erection even in the absence of sexual desire, providing a distinct advantage for certain patients.^13^ Additionally, while alprostadil is generally more expensive than PDE5i on a global scale,^43^ its use is particularly beneficial for patients for whom PDE5i are contraindicated, ineffective, or not tolerated.^44^

Furthermore, testosterone plays a key role in regulating PDE5 expression in the corpus cavernosum, and low testosterone levels can lead to diminished responses to PDE5 inhibitors.^45^ Available evidence suggests that testosterone supplementation can enhance the efficacy of PDE5 inhibitors in hypogonadal men, making combination therapy a common strategy in such cases.^46^ On the other hand, the testosterone‐independent mechanism of testosterone makes it an effective treatment modality for men with ED who are hypogonadal or unresponsive to PDE5 inhibitors.^47^ Despite these advantages of alprostadil, previous studies have demonstrated that compliance with PD5E inhibitors is significantly higher, whereas adherence to alprostadil treatment remains notably lower.^48^

The differences between the topical and intraurethral administration routes have not been rigorously studied, however, the 2019 study by Cai et al. demonstrated that intraurethral administration was more effective in treating ED symptoms compared to topical application.^33^ Cai et al. suggested that the increased efficacy of intraurethral application could be attributed to less drug wastage.

In the present systematic review and meta‐analysis, we identified two studies that compared intraurethral alprostadil to intracavernosal injections.^26^, ^27^ These studies found that intracavernosal administration was more effective in treating ED symptoms and achieving firmer erections. However, the first study reported that the conventional administration combined with the lower rates of urogenital pain after intraurethral application made this way of application preferable compared to intracavernosal administration despite the lower efficacy.^27^ To complicate things further, the second study found that patients and their partners preferred intracavernosal injections despite the inconvenience of administration due to their high efficacy.^26^ Therefore, it seems that both ways of administration should be discussed with the patients and the decision on the preferred treatment modality should be based on the patients’ preferences.

While our study benefits from a robust methodological approach, it is essential to acknowledge certain limitations. The high levels of heterogeneity observed suggest variability across studies, which predominantly stems from the differences in study design, patient populations, preferred treatment protocols, across the included studies, discrepancies in the number of patients between treatment arms, as well as from the small number of available studies, and the relatively older data included in our systematic review and meta‐analysis. Consequently, our results should be interpreted with caution due to the low methodological quality of some included studies, which downgrade the level of provided evidence. Furthermore, in studies involving different alprostadil dosages, we merged the entire cohort. While this approach increased the sample size, it may also limit the generalizability of our findings, since higher dosages of topical or intraurethral alprostadil may be more effective. Given the scarcity of available data, we could not perform further subgroup or sensitivity analyses. Importantly, the limited number of included studies (less than five) in the meta‐analysis prevented us from conducting a publication bias analysis. Of note, it should be acknowledged that we imputed some missing SDs based on the correlation coefficients reported from other included studies. Although this method is recommended by the Cochrane Collaboration, the latter might have affected our outcomes. It should be also stressed that due to lack of follow‐up data beyond the treatment period in all identified trials, the long‐term safety and efficacy of topical and intraurethral alprostadil could not be explored. Lastly, most trials included in this systematic review and meta‐analysis are dated, and the scarcity of recent studies on the matter should be also considered as an important limitation of our study.

## 5 CONCLUSIONS

Our systematic review and meta‐analysis demonstrate that both topical and intraurethral alprostadil significantly improve erectile function in patients with ED compared to placebo. Despite these promising findings, the results should be interpreted with caution due to the important limitations of the present study. Both treatment modalities present effective alternatives for patients who do not respond to or cannot use PDE5i, offering a favourable safety profile with minimal AEs. Overall, further high‐quality, large‐scale RCTs are needed to confirm these results and establish more definitive clinical guidelines for the use of alprostadil in the management of ED.

## AUTHOR CONTRIBUTIONS

All authors participated in the drafting, writing, and editing of the manuscript. Iason Papadopoulos and Nikolaos Pyrgidis had full access to all the data in the study and take responsibility for the integrity of the data and the accuracy of the data analysis.

## CONFLICT OF INTEREST STATEMENT

The authors declare no conflicts of interest.

## Supporting information



Supporting Information

## Data Availability

The data that support the findings of this study are available from the corresponding author upon reasonable request.

## References

[1] NIH Consensus Conference . Impotence: NIH consensus development panel on impotence. JAMA. 1993;270(1):83. doi:10.1001/jama.1993.03510010089036 8510302

[2] Salonia A , Bettocchi C , Capogrosso P , et al. EAU Guidelines on Sexual and Reproductive Health 2024. European Association of Urology Guidelines 2024 Edition. European Association of Urology Guidelines Office; 2024.

[3] Braun M , Wassmer G , Klotz T , Reifenrath B , Mathers M , Engelmann U . Epidemiology of erectile dysfunction: results of the “Cologne Male Survey”. Int J Impot Res. 2000;12(6):305‐311. doi:10.1038/sj.ijir.3900622 11416833

[4] Feldman HA , Goldstein I , Hatzichristou DG , Krane RJ , McKinlay JB . Impotence and its medical and psychosocial correlates: results of the Massachusetts Male Aging Study. J Urol. 1994;151(1):54‐61. doi:10.1016/s0022-5347(17)34871-1 8254833

[5] Corona G , Cucinotta D , Di Lorenzo G , et al. The Italian Society of Andrology and Sexual Medicine (SIAMS), along with ten other Italian Scientific Societies, guidelines on the diagnosis and management of erectile dysfunction. J Endocrinol Invest. 2023;46(6):1241‐1274. doi:10.1007/s40618-023-02015-5 36698034 PMC9876440

[6] Pyrgidis N , Mykoniatis I , Haidich AB , et al. The effect of phosphodiesterase‐type 5 inhibitors on erectile function: an overview of systematic reviews. Front Pharmacol. 2021;12:735708. doi:10.3389/fphar.2021.735708 34557099 PMC8452927

[7] Munk NE , Knudsen JS , Comerma‐Steffensen S , Simonsen U . Systematic review of oral combination therapy for erectile dysfunction when phosphodiesterase type 5 inhibitor monotherapy fails. Sex Med Rev. 2019;7(3):430‐441. doi:10.1016/j.sxmr.2018.11.007 30711478

[8] Mykoniatis I , Pyrgidis N , Sokolakis I , et al. Assessment of Combination therapies vs monotherapy for erectile dysfunction: a systematic review and meta‐analysis. JAMA Netw Open. 2021;4(2):e2036337. doi:10.1001/jamanetworkopen.2020.36337 33599772 PMC7893498

[9] Urciuoli R , Cantisani TA , Carlini M , Giuglietti M , Botti FM . Prostaglandin E1 for treatment of erectile dysfunction. Cochrane multiple sclerosis and rare diseases of the CNS group. Cochrane Database Syst Rev. doi:10.1002/14651858.CD001784.pub2. Published online April 19, 2004. 15106162

[10] Hanchanale V , Eardley I . Alprostadil for the treatment of impotence. Expert Opin Pharmacother. 2014;15(3):421‐428. doi:10.1517/14656566.2014.873789 24369066

[11] Porst H . The rationale for prostaglandin E1 in erectile failure: a survey of worldwide experience. J Urol. 1996;155(3):802‐815. 8583582

[12] Weiss JN , Badlani GH , Ravalli R , Brettschneider N . Reasons for high drop‐out rate with self‐injection therapy for impotence. Int J Impot Res. 1994;6(3):171‐174. 7735362

[13] Costa P , Potempa AJ . Intraurethral alprostadil for Erectile dysfunction: a review of the literature. Drugs. 2012;72(17):2243‐2254. doi:10.2165/11641380-000000000-00000 23170913

[14] Moher D , Liberati A , Tetzlaff J , Altman DG , GroupPRISMA . Preferred reporting items for systematic reviews and meta‐analyses: the PRISMA statement. PLoS Med. 2009;6(7):e1000097. doi:10.1371/journal.pmed.1000097 19621072 PMC2707599

[15] Higgins JPT , Thomas J , Chandler J , eds. Cochrane Handbook for Systematic Reviews of Interventions. 1st ed. Wiley; 2019. doi:10.1002/9781119536604 PMC10284251 31643080

[16] Wan X , Wang W , Liu J , Tong T . Estimating the sample mean and standard deviation from the sample size, median, range and/or interquartile range. BMC Med Res Methodol. 2014;14:135. doi:10.1186/1471-2288-14-135 25524443 PMC4383202

[17] Sterne JAC , Savović J , Page MJ , et al. RoB 2: a revised tool for assessing risk of bias in randomised trials. BMJ. 2019;366:l4898. doi:10.1136/bmj.l4898 31462531

[18] Sterne JA , Hernán MA , Reeves BC , et al. ROBINS‐I: a tool for assessing risk of bias in non‐randomised studies of interventions. BMJ. 2016;355:i4919. doi:10.1136/bmj.i4919 27733354 PMC5062054

[19] Garrido‐Abad P , Senra‐Bravo I , Manfredi C , et al. Combination therapy with topical alprostadil and phosphodiesterase‐5 inhibitors after failure of oral therapy in patients with erectile dysfunction: a prospective, two‐arm, open‐label, non‐randomized study. Int J Impot Res. 2022;34(2):164‐171. doi:10.1038/s41443-020-00400-9 33483603

[20] Buvat J , Damaj B , Fernando Y , Frank D , Burger M , Moncada I . Clinically Significant Improvement of Erectile Function Following Treatment with Alprostadile Cream (Vitaros) in 1651 Patients with Erectile Dysfunction. ESSM; 2012.

[21] Goldstein I , Payton TR , Schechter PJ . A double‐blind, placebo‐controlled, efficacy and safety study of topical gel formulation of 1% alprostadil (Topiglan) for the in‐office treatment of erectile dysfunction. Urology. 2001;57(2):301‐305. doi:10.1016/S0090-4295(00)00936-5 11182341

[22] McVary KT , Polepalle S , Riggi S , Pelham RW . Topical prostaglandin E1 SEPA gel for the treatment of erectile dysfunction. J Urol. 1999;162(3 Pt 1):726‐730. discussion 730–731. doi:10.1097/00005392-199909010-00025 10458353

[23] Padma‐Nathan H , Steidle C , Salem S , Tayse N , Yeager J , Harning R . The efficacy and safety of a topical alprostadil cream, Alprox‐TD®, for the treatment of erectile dysfunction: two phase 2 studies in mild‐to‐moderate and severe ED. Int J Impot Res. 2003;15(1):10‐17. doi:10.1038/sj.ijir.3900940 12605235

[24] Padma‐Nathan H , Yeager JL . An integrated analysis of alprostadil topical cream for the treatment of erectile dysfunction in 1732 patients. Urology. 2006;68(2):386‐391. doi:10.1016/j.urology.2006.02.027 16904458

[25] Belinski C . Evaluation of combined pharmacologic treatment involving tadalafil and alprostadil intraurethral gel for penile rehabilitation after radical prostatectomy. Farmacia. 2021;69(3):540‐547. doi:10.31925/farmacia.2021.3.17

[26] Shabsigh R , Padma‐Nathan H , Gittleman M , McMurray J , Kaufman J , Goldstein I . Intracavernous alprostadil alfadex is more efficacious, better tolerated, and preferred over intraurethral alprostadil plus optional ACTIS: a comparative, randomized, crossover, multicenter study. Urology. 2000;55(1):109‐113. doi:10.1016/S0090-4295(99)00442-2 10654905

[27] Shokeir AA , Alserafi MA , Mutabagani H . Intracavernosal versus intraurethral alprostadil: a prospective randomized study. BJU Int. 1999;83(7):812‐815. doi:10.1046/j.1464-410x.1999.00021.x 10368203

[28] Coca V . P‐02‐14 Intra‐urethral alprostadil gel usage in male sexual dysfunction restores not only erectile performances but improves also the conjunct premature ejaculation. J Sex Med. 2020;17(Supplement_2):S176‐S176. doi:10.1016/j.jsxm.2020.04.169

[29] McCullough AR , Hellstrom WG , Wang R , Lepor H , Wagner KR , Engel JD . Recovery of erectile function after nerve sparing radical prostatectomy and penile rehabilitation with nightly intraurethral alprostadil versus sildenafil citrate. J Urol. 2010;183(6):2451‐2456. doi:10.1016/j.juro.2010.01.062 20403617

[30] Hellstrom WJG , Bennett AH , Gesundheit N , et al. A double‐blind, placebo‐controlled evaluation of the erectile response to transurethral alprostadil. Urology. 1996;48(6):851‐856. doi:10.1016/S0090-4295(96)00428-1 8973666

[31] Padma‐Nathan H , Lue TF , Shabsigh R . Treatment of men with erectile dysfunction with transurethral alprostadil. N Engl J Med. 1997;336(1):1‐7. 8970933 10.1056/NEJM199701023360101

[32] Williams G , Abbou CC , Amar ET , et al. Efficacy and safety of transurethral alprostadil therapy in men with erectile dysfunction. MUSE Study Group. Br J Urol. 1998;81(6):889‐894. doi:10.1046/j.1464-410x.1998.00703.x 9666777

[33] Cai T , Palumbo F , Liguori G , et al. The intra‐meatal application of alprostadil cream (Vitaros®) improves drug efficacy and patient's satisfaction: results from a randomized, two‐administration route, cross‐over clinical trial. Int J Impot Res. 2019;31(2):119‐125. doi:10.1038/s41443-018-0087-6 30323234

[34] McGuinness LA , Higgins JPT . Risk‐of‐bias VISualization (robvis): an R package and Shiny web app for visualizing risk‐of‐bias assessments. Res Synth Methods. 2020;12(1):55‐61. doi:10.1002/jrsm.1411 32336025

[35] Moncada I , Cuzin B . Clinical efficacy and safety of Vitaros ^©^ /Virirec ^©^ (alprostadil cream) for the treatment of erectile dysfunction. Urologia. 2015;82(2):84‐92. doi:10.5301/uro.5000116 25744707

[36] Yeager J , Beihn RM . Retention and migration of alprostadil cream applied topically to the glans meatus for erectile dysfunction. Int J Impot Res. 2005;17(1):91‐95. doi:10.1038/sj.ijir.3901285 15538395

[37] Carvalheira AA , Pereira NM , Maroco J , Forjaz V . Dropout in the treatment of erectile dysfunction with PDE5: a study on predictors and a qualitative analysis of reasons for discontinuation. J Sex Med. 2012;9(9):2361‐2369. doi:10.1111/j.1743-6109.2012.02787.x 22616766

[38] Shamloul R , Ghanem H . Erectile dysfunction. Lancet. 2013;381(9861):153‐165. doi:10.1016/S0140-6736(12)60520-0 23040455

[39] Jannini EA , Sternbach N , Limoncin E , et al. Health‐related characteristics and unmet needs of men with erectile dysfunction: a survey in five European countries. J Sex Med. 2014;11(1):40‐50. doi:10.1111/jsm.12344 24314303

[40] Moncada I , Martinez‐Salamanca J , Ruiz‐Castañe E , Romero J . Combination therapy for erectile dysfunction involving a PDE5 inhibitor and alprostadil. Int J Impot Res. 2018;30(5):203‐208. doi:10.1038/s41443-018-0046-2 30050072

[41] Anaissie J , Hellstrom WJ . Clinical use of alprostadil topical cream in patients with erectile dysfunction: a review. Res Rep Urol. 2016;8:123‐131. doi:10.2147/RRU.S68560 27536559 PMC4977016

[42] Becher E . Topical alprostadil cream for the treatment of erectile dysfunction. Expert Opin Pharmacother. 2004;5(3):623‐632. doi:10.1517/14656566.5.3.623 15013930

[43] Moses RA , Anderson RE , Kim J , et al. Erectile dysfunction management after failed phosphodiesterase‐5‐inhibitor trial: a cost‐effectiveness analysis. Transl Androl Urol. 2019;8(4):387‐394. doi:10.21037/tau.2019.03.10 31555563 PMC6732088

[44] Cuzin B . Alprostadil cream in the treatment of erectile dysfunction: clinical evidence and experience. Ther Adv Urol. 2016;8(4):249‐256. doi:10.1177/1756287216644116 27928427 PMC5131739

[45] Zhang XH , Morelli A , Luconi M , et al. Testosterone regulates PDE5 expression and in vivo responsiveness to tadalafil in rat corpus cavernosum. Eur Urol. 2005;47(3):409‐416. discussion 416. doi:10.1016/j.eururo.2004.10.021 15716209

[46] Greco EA , Spera G , Aversa A . Combining testosterone and PDE5 inhibitors in erectile dysfunction: basic rationale and clinical evidences. Eur Urol. 2006;50(5):940‐947. doi:10.1016/j.eururo.2006.06.049 16979814

[47] Shabsigh R . Hypogonadism and erectile dysfunction: the role for testosterone therapy. Int J Impot Res. 2003;15(Suppl 4):S9‐S13. doi:10.1038/sj.ijir.3901030 12934045

[48] Hedelin H , Ströberg P . Treatment for erectile dysfunction based on patient‐reported outcomes: to every man the PDE5 inhibitor that he finds superior. Drugs. 2005;65(16):2245‐2251. doi:10.2165/00003495-200565160-00001 16266193

