# Supplementary material for: The effect of topical and intraurethral alprostadil on erectile function: A systematic review and meta‐analysis
Source: Andrology. 2025 Mar 19;13(8):2235–45. doi: 10.1111/andr.70025 (PMC12569735; doi:10.1111/andr.70025)
Supplement: Supplementary file 1 — Supporting Information [file ANDR-13-2235-s001.docx]

Data Supplement 1: PubMed search syntax and search string

**Search syntax**

**ID Search**

#1 "Vitaros " [All fields]

#2 "MUSE" [All fields]

#3 "Alprostadil" [All fields]

#4 OR #1-3

#5 “Sexual” [All fields]

#6 “Erectile” [All fields]

#7 “Impotence” [All fields]

#8 “Erection” [All fields]

#9 OR #5-8

#10 #4 AND #9

**Search string**

("Vitaros"[All Fields] OR "MUSE"[All Fields] OR "Alprostadil"[All Fields]) AND ("Sexual"[All Fields] OR "Erectile"[All Fields] OR "Impotence"[All Fields] OR "Erection"[All Fields])

The search strategy was developed for PubMed and modified accordingly for the other databases.

Data Supplement 2: References of all excluded studies with reasons for exclusion

**Overlapping study records**

1. Costabile RA, Spevak M, Fishman IJ, Govier FE, Hellstrom WJ, Shabsigh R, et al. Efficacy and safety of transurethral alprostadil in patients with erectile dysfunction following radical prostatectomy. J Urol. 1998;160(4):1325-1328.
2. McCullough AR, Bytyci A, Goodwin B, Alukal J. Acute changes in penile oximetry after single utilization of the vacuum erection device (VED) compared to intraurethral alprostadil (IUA) and intracavernosal injections (ICI) in patients following radical prostatectomy (RP). J Urol. 2009;181(4S):486-487. https://doi.org/10.1016/S0022-5347(09)61377-X.
3. Mulhall J, Buvat J, Goldstein I, Damaj B, Frank D, Fernando Y. Vitaros® efficacy and safety in Viagra® non-responders with longer-term use. J Urol. 2013;189(4S). https://doi.org/10.1016/j.juro.2013.02.2997.
4. Porst H. Transurethral application of alprostadil with MUSE™ (medicated urethral system for erection). Actual overview and personal experiences. Der Urologe A. 1998;37(4):410-416. https://doi.org/10.1007/s001200050199.
5. Steidle C, Padma-Nathan H, Salem S, Tayse N, Thwing D, Fendl J, Yeager J, Harning R. Topical alprostadil cream for the treatment of erectile dysfunction: A combined analysis of the phase II program. Urology. 2002;60(6):1077-1082. https://doi.org/10.1016/S0090-4295(02)01980-5.
6. Williams G, Abbou C, Amar E, Desvaux P, Flam T, Lycklama À Nijeholt AA, Lynch J, et al. The effect of transurethral alprostadil on the quality of life of men with erectile dysfunction, and their partners. Br J Urol. 1998;82(6):847-854. https://doi.org/10.1046/j.1464-410X.1998.00937.x.

**No Alprostadil as Active Treatment**

1. Elhardt PF, Eng E, Plas E, Hu Bner WA. Comparison of intraurethral liposomal and intracavernosal prostaglandin-E1 in the management of erectile dysfunction. Br J Urol. 1998.
2. Jiang H, Xu QQ, Hong K, Wang XF, Zhu JC. [Efficacy and safety of PGE1 cream in the treatment of erectile dysfunction]. Zhonghua Nan Ke Xue. 2003;9(2):97-99.

**Studies not reporting clinical outcomes of interest**

1. Auerbach S, Goldstein I, Damaj B, Frank D, Hachicha M, Schupp J. Integrated efficacy results of two phase 3 clinical trials for male erectile dysfunction (ED) using Vitaros, a topical alprostadil cream with subgroup analyses. J Sex Med. 2012;9:47-48. doi:10.1111/j.1743-6109.2012.02633.x. Available from: http://ovidsp.ovid.com/ovidweb.cgi?T=JS&PAGE=reference&D=emed13&NEWS=N&AN=70664442.
2. Porst H. Transurethral alprostadil with MUSE (medicated urethral system for erection) vs intracavernous alprostadil--a comparative study in 103 patients with erectile dysfunction. Int J Impot Res. 1997;9(4):187-192. doi:10.1038/sj.ijir.3900318

**Single arm, non-comparative studies**

1. Tal R, Bennett NE, Stember DS, Katz DJ, Narus JB, Martelli A, et al. Erectile hemodynamics assessment in men with persistent erectile dysfunction after 5-alpha reductase inhibitor use. J Urol [Internet]. 2012 Apr [cited 2024 Jul 7];187(4S). Available from: http://www.jurology.com/doi/10.1016/j.juro.2012.02.1263.
2. Ahn H, Lee S, Yoon S, Hann H, Hong J. A comparison of colour duplex ultrasonography after transurethral alprostadil and intracavernous alprostadil in the assessment of erectile dysfunction. J Int Med Res. 2004 May;32(3):317-323.
3. Mulhall JP, Jahoda AE, Ahmed A, Parker M. Analysis of the consistency of intraurethral prostaglandin E1 (MUSE) during at-home use. Urology. 2001 Aug;58(2):262-266.
4. Guay AT, Perez JB, Velásquez E, Newton RA, Jacobson JP. Clinical experience with intraurethral alprostadil (MUSE®) in the treatment of men with erectile dysfunction. Eur Urol. 2000;38(6):671-676.
5. Fulgham PF, Cochran JS, Feagins BA, Gross MB, Kadesky KT, Kadesky MC, et al. Disappointing initial results with transurethral alprostadil for erectile dysfunction in a urology practice setting.
6. Thomas C, Kartsaklis P, Konstantinidis C, Louka G. Efficacy and safety of treatment with the use of intraurethral alprostadil (Vitaros©) on demand in men with spinal cord injury and multiple sclerosis. Eur Urol Open Sci. 2023 Nov;57
7. Garrido Abad P, Sinués Ojas B, Martínez Blázquez L, Conde Caturla P, Fernández Arjona M. Eficacia y seguridad del alprostadil intrauretral en pacientes con disfunción eréctil refractarios al tratamiento mediante inhibidores de la 5-fosfodiesterasa. Actas Urol Esp. 2015 Dec;39(10):635-640.
8. Bodner DR, Haas CA, Krueger B, Seftel AD. Intraurethral alprostadil for treatment of erectile dysfunction in patients with spinal cord injury. Urology. 1999 Jan;53(1):199-202.
9. Potempa AJ, Potempa D, Görlich H, Stolpmann R. Intraurethral zu applizierendes alprostadil zur behandlung der primär organisch bedingten erektilen dysfunktion in der praxis. Arzneimittelforschung. 2011 Dec 21;57(06):299-308.
10. Raina R, Agarwal A, Ausmundson S, Mansour D, Zippe CD. Long-term efficacy and compliance of MUSE for erectile dysfunction following radical prostatectomy: SHIM (IIEF-5) analysis. Int J Impot Res. 2005 Jan;17(1):86-90.
11. Rooney M, Pfister W, Mahoney M, Nelson M, Yeager J, Steidle C. Long-term, multicenter study of the safety and efficacy of topical alprostadil cream in male patients with erectile dysfunction. J Sex Med. 2009 Feb;6(2):520-534.
12. Kim S, Ahn T, Choi H, Choi N, Chung T, Chung W, et al. Multicenter study of the treatment of erectile dysfunction with transurethral alprostadil (MUSE) in Korea. Int J Impot Res. 2000 Apr;12(2):97-101.
13. Werthman P, Rajfer J. Muse therapy: preliminary clinical observations. Urology. 1997 Nov;50(5):809-811.
14. Khan MA, Raistrick M, Mikhailidis DP, Morgan RJ. MUSE™: clinical experience. Curr Med Res Opin. 2002 Jan;18(2):64-67.
15. Ekman P, Sjögren L, Englund G, Persson B-E. Optimizing the therapeutic approach of transurethral alprostadil. BJU Int. 2000 Jul;86(1):68-74.
16. Della Camera PA, Cito G, Morselli S, Tasso G, Laruccia N, Cocci A, et al. Vitaros for the treatment of post prostatectomy erectile dysfunction. An effective alternative? J Sex Med. 2017 Apr;14
17. Thomas C, Poulakis V. Could Vitaros™ be considered as a viable alternative for the treatment of post-prostatectomy erectile dysfunction? A prospective study from a single center. J Sex Med. 2019 May;16
18. Nehra A, Blute ML, Barrett DM, Moreland RB. Rationale for combination therapy of intraurethral prostaglandin E1 and sildenafil in the salvage of erectile dysfunction patients desiring noninvasive therapy. Int J Impot Res. 2002 Feb;14(S1)
19. Raina R, Pahlajani G, Agarwal A, Zippe CD. The early use of transurethral alprostadil after radical prostatectomy potentially facilitates an earlier return of erectile function and successful sexual activity. *BJU Int*. 2007;100(6):1317-1321. doi:10.1111/j.1464-410X.2007.07124.x.
20. Chiang HS, Wen TC, Liang JF. Titration study of MUSE (Medicated Urethral System for Erection) in erectile dysfunction. *J Formos Med Assoc*. 2000;99(12):926-930.
21. Della Camera PA, Morselli S, Cito G, et al. Topical alprostadil (Vitaros^©^) in the treatment of erectile dysfunction after non-nerve-sparing robot-assisted radical prostatectomy. *Urologia*. 2018;85(2):55-59. doi:10.5301/uj.5000267.
22. Kim ED, McVary KT. Topical prostaglandin-E1 for the treatment of erectile dysfunction. *J Urol*. 1995;153(6):1828-1830.
23. Engel JD, McVary KT. Transurethral alprostadil as therapy for patients who withdrew from or failed prior intracavernous injection therapy. Urology. 1998 May;51(5):687-692.
24. Li MK, Lim PHC, Wong MYC, Fok A, Adaikan PG, et al. Transurethral alprostadil for the treatment of erectile dysfunction: results of a multicentre trial.
25. Jaffe JS, Antell MR, Greenstein M, Ginsberg PC, Mydlo JH, Harkaway RC. Use of intraurethral alprostadil in patients not responding to sildenafil citrate. Urology. 2004 May;63(5):951-954.

**Not Relevant to our Research Question**

1. Mantovani F. Alprostadil plus vacuum (VITARUM) in severe erectile dysfunction (ED). Arch Ital Urol Androl. 2017;89(2):146.
2. Kim JM, Joh YD, Huh JD, Choi S. Doppler sonography of the penile cavernosal artery: Comparison of intraurethral instillation and intracorporeal injection of prostaglandin E1. J Clin Ultrasound. 2001;29(5):273-278.
3. Khera M, Mohamed O, Colen JS, Link RE, Miles BJ, Lipshultz LI. Erectile preservation following radical prostatectomy: The Baylor experience. J Urol. 2009;181(4):524. doi:10.1016/j.juro.2009.02.1263. Available from: http://ovidsp.ovid.com/ovidweb.cgi?T=JS&PAGE=reference&D=emed11&NEWS=N&AN=70113563.
4. Peterson CA, Bennett AH, Hellstrom WJ, Kaiser FE, Morley JE, Nemo KJ, et al. Erectile response to transurethral alprostadil, prazosin and alprostadil-prazosin combinations. J Urol. 1998;159(5):1523-1527; discussion 1527-1528.
5. Risi O, Manfredi A, Manica M, Lisanti RC, La Croce G, Saccà A. Intracavernosal versus intraurethral alprostadil before penile duplex ultrasound: Results from a comparative study. Eur Urol Suppl. 2018;17(2).
6. Benevides MD, Carson CC. Intraurethral application of alprostadil in patients with failed inflatable penile prosthesis. J Urol. 2000;163(3):785-787.
7. John H, Lehmann K, Hauri D. Intraurethral prostaglandin improves quality of vacuum erection therapy. Eur Urol. 1996;29(2):224-226.
8. Yeager J, Beihn RM. Retention and migration of alprostadil cream applied topically to the glans meatus for erectile dysfunction. Int J Impot Res. 2005;17(1):91-95.
9. Lombardo T, Giammusso B, Frontini V, D’Arpa S, Pafumi C, Caruso S. Thalassaemic men affected by erectile dysfunction treated with transurethral alprostadil. [Journal name]. [Publication date];[Volume(Issue)]:[Page numbers].
10. Lewis RW. Transurethral alprostadil with MUSE (medicated urethral system for erection) vs intracavernous alprostadil--a comparative study in 103 patients with erectile dysfunction. Int J Impot Res. 1998;10(1):61-62.

Data Supplement 3: Risk of bias in RCTs


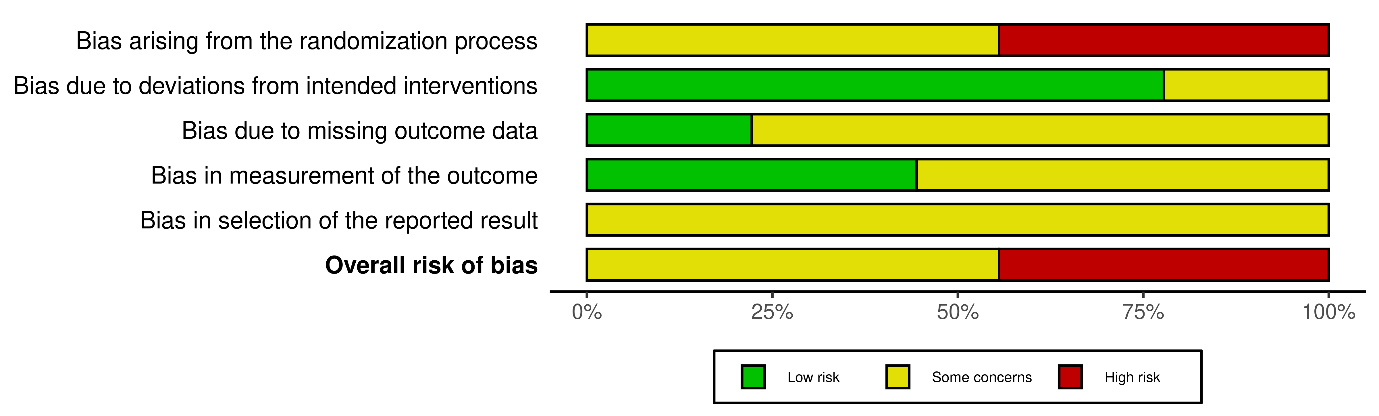


**Data Supplement 3.1**: Overall risk of bias assessment in included RCTs.


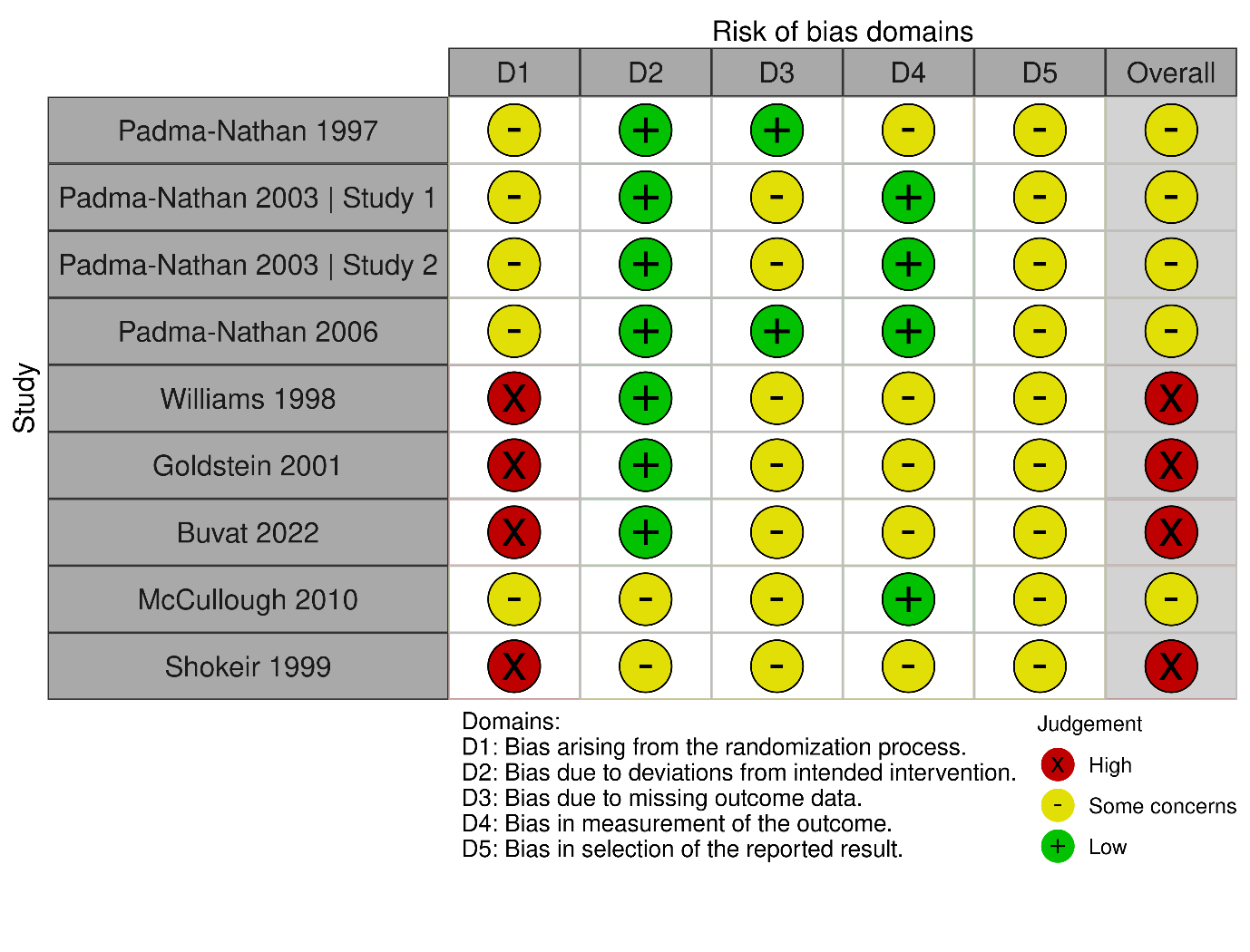


**Data Supplement 3.2**: Study-by-study risk of bias assessment in included RCTs.

Data Supplement 4: Risk of bias in non-RCTs


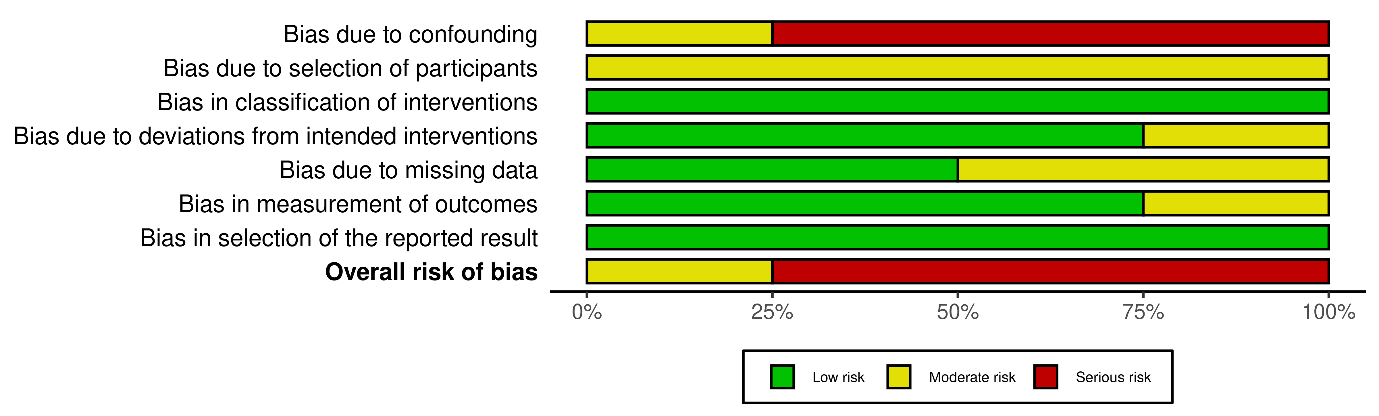


**Data Supplement 4.1**: Overall risk of bias assessment in included non-RCTs.


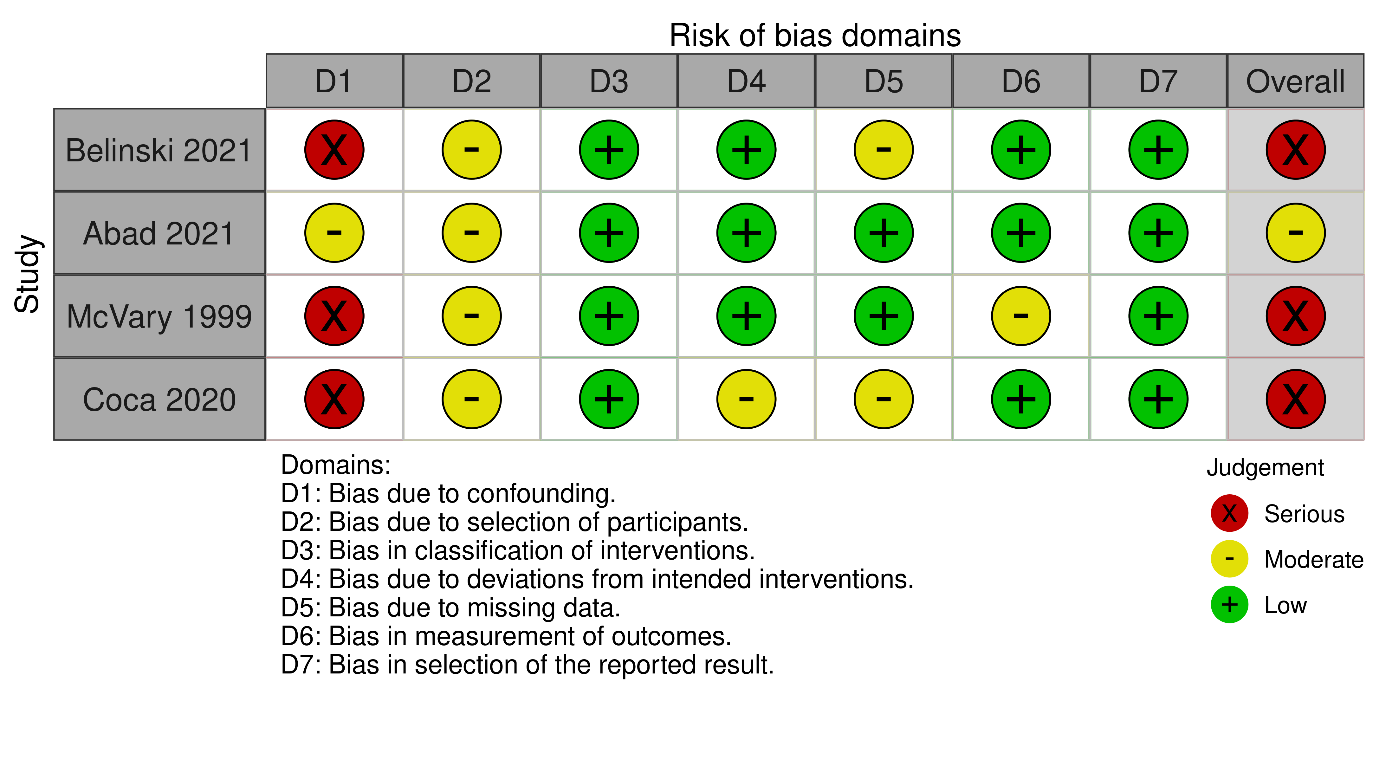


**Data Supplement 4.2**: Study-by-study risk of bias assessment in included non-RCTs.
